# A *Shigella* species variant is causally linked to intractable functional constipation

**DOI:** 10.1172/JCI150097

**Published:** 2022-07-15

**Authors:** Xin Chen, Tian-Tian Qiu, Ye Wang, Li-Yang Xu, Jie Sun, Zhi-Hui Jiang, Wei Zhao, Tao Tao, Yu-Wei Zhou, Li-Sha Wei, Ye-Qiong Li, Yan-Yan Zheng, Guo-Hua Zhou, Hua-Qun Chen, Jian Zhang, Xiao-Bo Feng, Fang-Yu Wang, Ning Li, Xue-Na Zhang, Jun Jiang, Min-Sheng Zhu

**Affiliations:** 1 State Key Laboratory of Pharmaceutical Biotechnology, Jiangsu Key Laboratory of Molecular Medicine and; 2 Research Institute of General Surgery, Jinling Hospital, Medical School of Nanjing University, Nanjing, China.; 3 Shaanxi An-Ning-Yunsheng Biotechnology Limited Company, Xi’an, China.; 4 Department of Clinical Pharmacy, Jinling Hospital, Medical School of Nanjing University, Nanjing, China.; 5 School of Life Science, Nanjing Normal University, Nanjing, China.; 6 State Key Laboratory for Conservation and Utilization of Bio-Resources, School of Life Sciences, Center for Life Sciences, Yunnan University, Kunming, China.; 7 Department of Gastroenterology and Hepatology, Jinling Hospital, Medical School of Nanjing University, Nanjing, China.

**Keywords:** Gastroenterology, Bacterial infections

## Abstract

Intractable functional constipation (IFC) is the most severe form of constipation, but its etiology has long been unknown. We hypothesized that IFC is caused by refractory infection by a pathogenic bacterium. Here, we isolated from patients with IFC a *Shigella* species — peristaltic contraction–inhibiting bacterium (PIB) — that significantly inhibited peristaltic contraction of the colon by production of docosapentenoic acid (DPA). PIB colonized mice for at least 6 months. Oral administration of PIB was sufficient to induce constipation, which was reversed by PIB-specific phages. A mutated PIB with reduced DPA was incapable of inhibiting colonic function and inducing constipation, suggesting that DPA produced by PIB was the key mediator of the genesis of constipation. PIBs were detected in stools of 56% (38 of 68) of the IFC patients, but not in those of non-IFC or healthy individuals (0 of 180). DPA levels in stools were elevated in 44.12% (30 of 68) of the IFC patients but none of the healthy volunteers (0 of 97). Our results suggest that *Shigella* sp. PIB may be the critical causative pathogen for IFC, and detection of fecal PIB plus DPA may be a reliable method for IFC diagnosis and classification.

## Graphical abstract

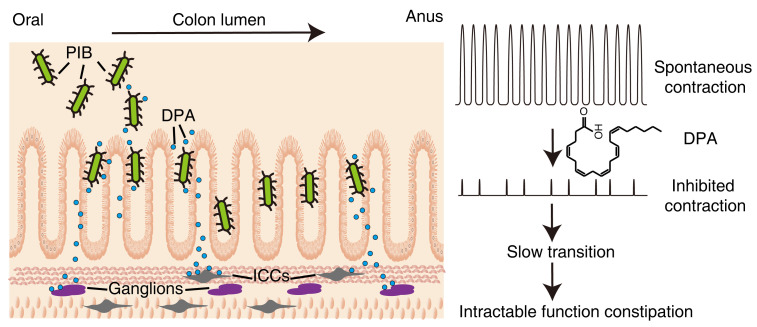

## Introduction

Chronic constipation (CC) is a common and multifactorial disorder characterized by reduced bowel movement, hard stools, and excessive fecal straining (1–3). CC may be primary (idiopathic) or associated with a number of disorders or medications (2). Based on clinical symptoms and anorectal evacuation, the primary CC may be diagnosed and classified by Rome criteria (III or IV) into functional constipation (FC) and constipation-predominant irritable bowel syndrome (IBS-C). Based on colonic and anorectal function, clinicians also classify CC into normal transit constipation, slow-transit constipation (STC), and pelvic floor dysfunction or defecatory disorders. After proper treatments — including lifestyle change, diet improvement, laxative usage, and prokinetic reagents (4–6) — CC can be cured in some patients (2–4). The defecatory disorders usually respond well to biofeedback-aided pelvic floor retraining and surgical procedures such as ileorectal anastomosis (2). However, about one-third of FC patients with STC do not respond to these treatments and experience repeated incidents, and such a medically refractory STC is considered an indication for colectomy (2, 7–10). To highlight its refractory nature, we here refer to the disorder in such patients in which conventional medications are ineffective as “intractable FC” (IFC). IFC is considered the most severe form of constipation, and its etiology remains unknown.

IFC pathology is characterized by slow transit due to reduced colonic motility or peristaltic movement. Several factors associated with slow transportation have been identified (1). In particular, recent whole-genome analysis and fecal transplantation studies have suggested that the gut microbiome may be an essential factor influencing gut transit and hence the CC phenotype (11–16). However, the key causative factor of the slow transit remains unclear. The nature of IFC phenotypes prompted us to hypothesize that IFC might be caused by refractory infection by certain bacteria with an inhibitory role in colon motility. Based on this hypothesis, we functionally screened individual bacterial colonies from IFC colonic mucosa through an ex vivo assay and identified a bacterial isolate — peristaltic contraction–inhibiting bacterium (PIB) — that could inhibit colonic contraction by producing the long-chain unsaturated fatty acid docosapentenoic acid (DPA). 16S rRNA and genomic analyses indicated that PIB was a species of *Shigella*. In in vivo experiments, PIBs entering the body via the mouth colonized mice and induced a constipation-like phenotype. Application of PIB-specific bacterial phages was sufficient to clear the colonized PIB and reverse the constipation phenotype. We established a PCR assay to detect PIB in human feces. About 56% of the patients with IFC were fecal PIB^+^, while all healthy individuals in the control group and non-IFC patients were PIB^–^. Fecal DPA levels were elevated in about 44% of the IFC patients. Our study reveals that PIB may serve as the pathogenic bacterium of IFC, a finding that is potentially critical for developing diagnostic and therapeutic methods for the disease.

## Results

### Identification of contraction-inhibiting bacteria in humans.

**Figure 1:**
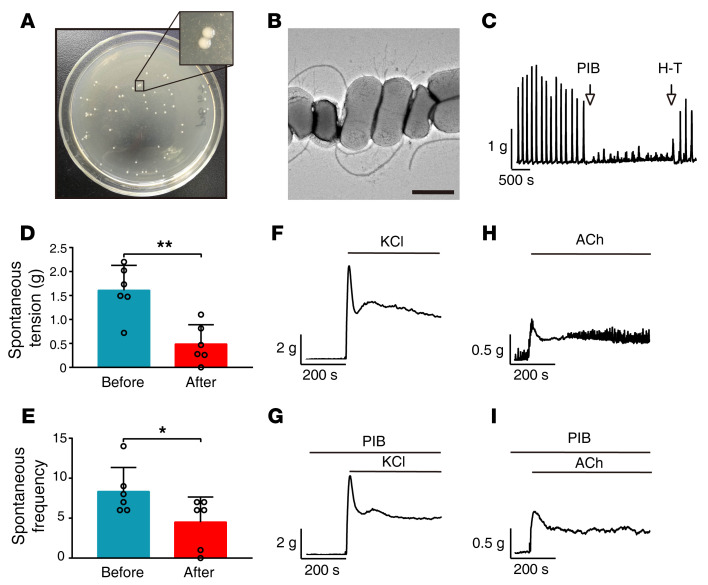
Identification of PIB from IFC patients. (**A**) Typical morphology of PIB colonies. (**B**) Individual PIB. Scale bar: 1 μm. (**C**) Representative contraction tracings before and after treatment with PIB culture supernatant. (**D** and **E**) Quantification of the contraction in **C** (*n* = 6). (**F**–**I**) Representative contraction tracings of PIB culture supernatant–pretreated colons after stimulation with 87 mM KCl (**F** and **G**) or 100 μM ACh (**H** and **I**). PIB culture supernatant was applied over 15 minutes before stimulating with KCl or ACh. The experiments were repeated at least 3 times. Data are presented as mean ± SD. *P < 0.05, **P < 0.01 (paired 2-tailed *t* test).

In order to isolate the pathogenic bacteria underlying constipation, we selected 4 patients with FC who were diagnosed according to Rome IV criteria. All the patients (1 male and 3 female, aged 25–52) showed indications of colectomy (slow transit, no response to conventional medications, strong desire for surgery, etc.) and were regarded as having IFC. Colon biopsies were collected from these patients and washed thoroughly with sterilized normal solution. To harvest more bacteria colonies, we cultured the wash solution on nutritious blood culture plates and inoculated the grown colonies in Luria broth (LB) medium individually. We first used segments of jejunum — which is typically characterized by good peristaltic contraction — to measure the inhibitory effects of culture supernatants on contraction. After initially screening thousands of colonies, we identified 3 with potent inhibitory effects on jejunum peristaltic contraction. To confirm the effects on the colon, we measured peristaltic contraction in colonic segments and observed that all 3 colonies had the same effect. These colonies presented a white, plump, and round morphology (Figure 1A), and the single bacterium had an apparent rod shape, a single flagellum, and multiple fimbriae (Figure 1B). As the 16S ribosome RNA gene sequences (rrs) of these bacterial colonies were found to have greater than 99.7% similarity, we considered the colonies identical (data not shown). As these bacteria are able to maintain their inhibitory effect for several passages, we refer to them as “peristaltic contraction–inhibiting bacteria” (PIB). Colonic contraction amplitude and frequency were significantly reduced after the addition of PIB culture supernatants (see Figure 1, C–E, for representative examples). As peristaltic contraction reflected the spontaneous contraction in the resting condition, we then tested the effect of PIB culture supernatants on colonic contraction with KCl or acetylcholine (ACh) stimulation. Upon incubation with the PIB culture supernatants, the colon showed a contraction response comparable to that of the control group (Figure 1, F–I). Thus, the PIB culture supernatant was capable of inhibiting the spontaneous as opposed to the stimulation-induced contraction.

### Genetic characterization of PIB.

**Figure 2:**
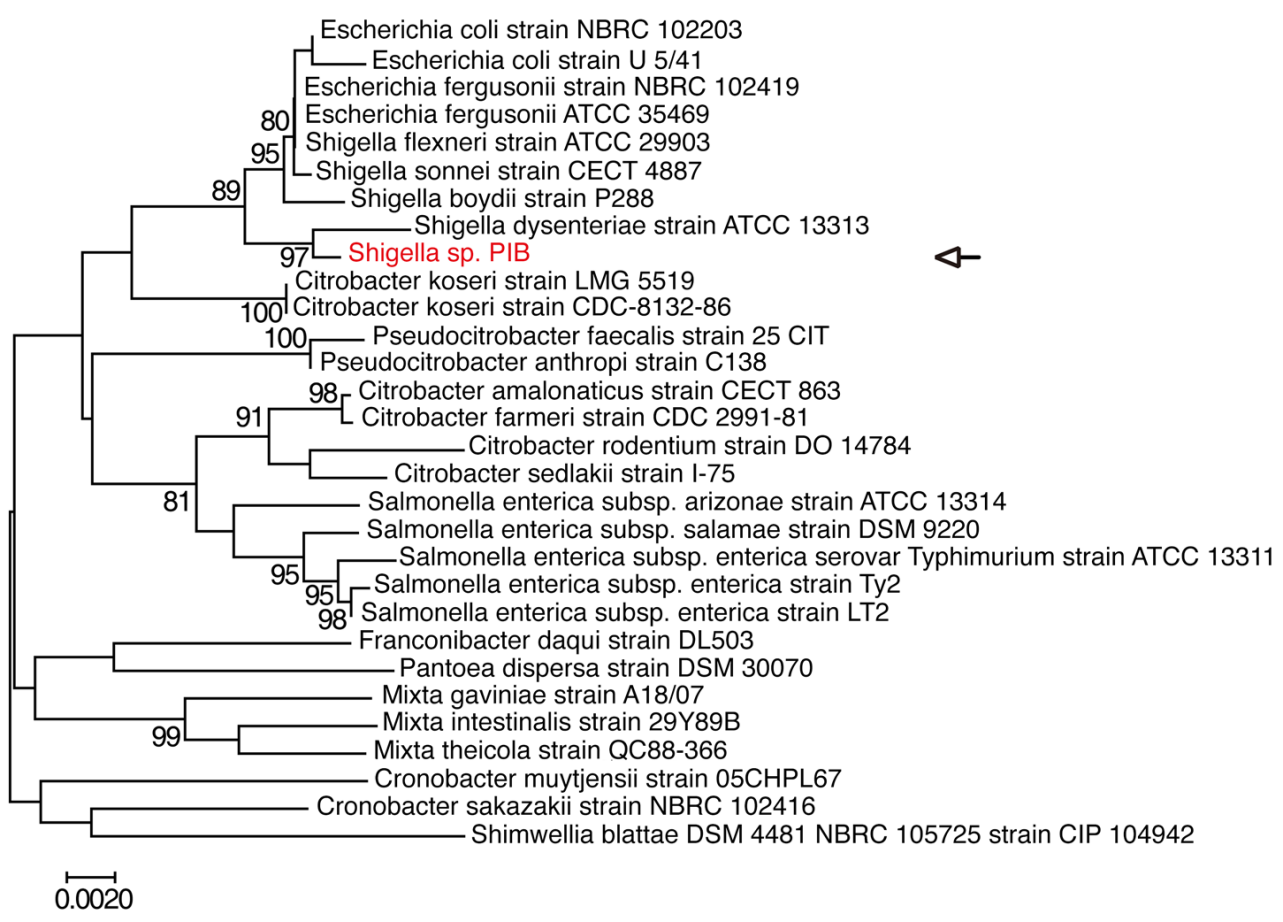
Phylogenetic relationship between PIB and related bacteria. A phylogenetic tree based on the PIB 16S rrs (1464 bp) was constructed by the neighbor-joining method, and the distance was calculated with the maximum composite likelihood method as the number of base substitutions per site. Bootstrap values (>80%) based on 1000 replications are listed on the branch node, and the branch length of the phylogenetic tree is proportional to the evolutionary distance. The 16S rrs genes of related bacteria were extracted from GenBank. Phylogenetic analysis was performed using Molecular Evolutionary Genetics Analysis software, version 7.0.

The PIB 16S rrs (1464 bp) was amplified by PCR and sequenced. Phylogenetic analysis showed that PIB 16S rrs was similar to that of the genus *Shigella*, with a high bootstrap value (97%). The evolutionary distance from the Shigella dysenteriae strain suggested that PIB is a novel strain of the genus Shigella (Figure 2). This was further supported by the PIB whole-genome sequence data (Table 1). The PIB genome contained more noncoding RNAs and fewer genes than other *Shigella* strains. The genome size of PIB also differed from that of other *Shigella* variants. We thus suggest that PIB is a new isolate of the genus *Shigella*.

### PIB inhibits colon contraction by producing DPA.

**Figure 3:**
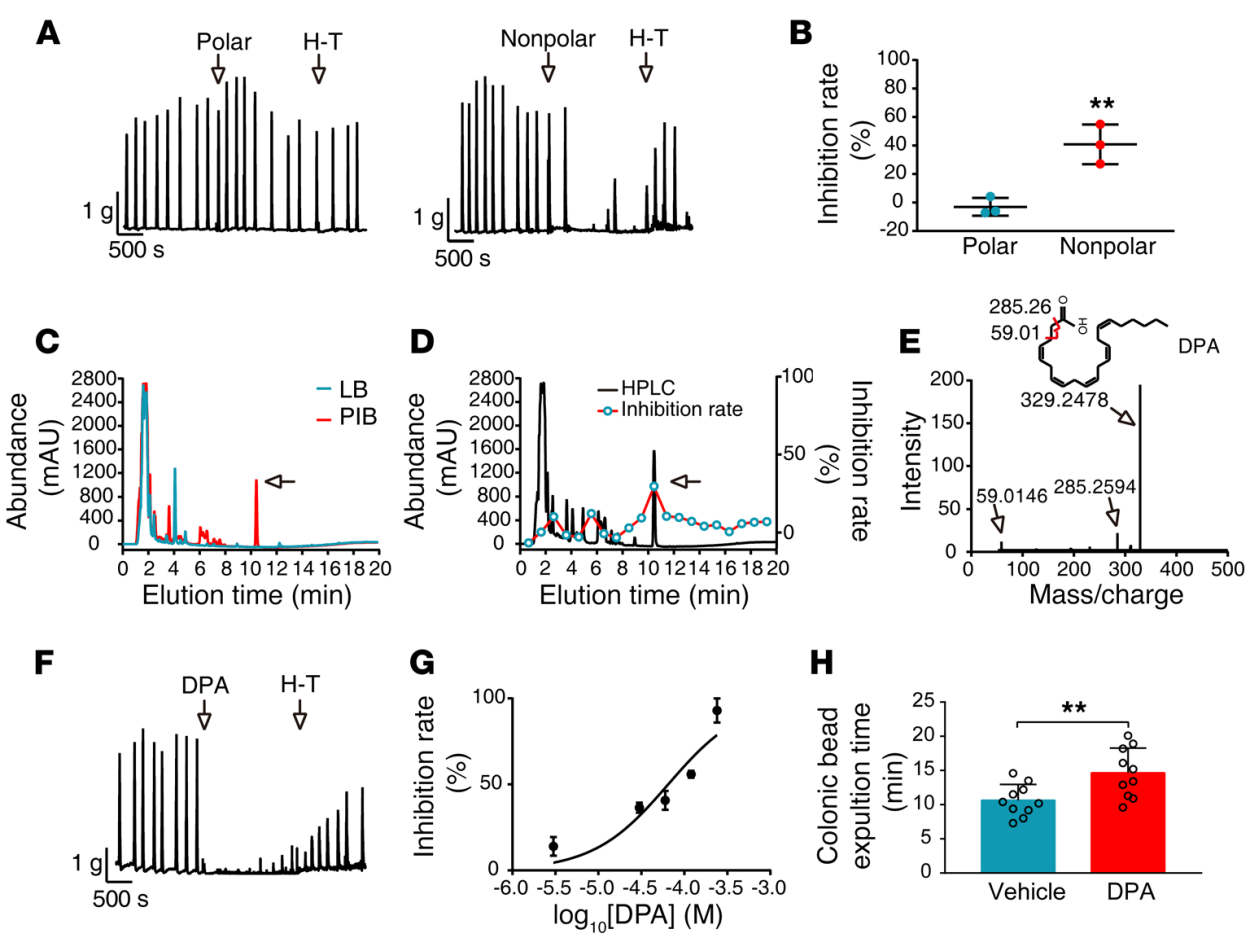
DPA is the active factor released from PIB. (**A** and **B**) PIB culture supernatant was extracted with methanol/H_2_O/dichloromethane, and the substances in polar and nonpolar phases were subjected to contraction measurement. The contraction inhibition rate of the substances in the 2 phases was calculated (*n* = 3). (**C**) The substances in the dichloromethane phase were analyzed with HPLC with a C18 column. The arrow indicates the extra peak (red) of PIB medium in contrast to LB medium. The experiments were repeated 3 times. (**D**) The contraction-inhibiting activity for each eluted fraction of HPLC was measured. The fraction about 10.2 minutes after elution showed the highest activity. (**E**) The major resulting fragment ions are indicated in mass spectra of active fraction peaks and were extracted as DPA. The numbers indicate molecular weights of fragment ions. (**F**) cDPA had a substantial inhibitory effect on contraction. (**G**) Quantitation of the inhibitory effect of DPA (*n* = 4). (**H**) Colonic transit test with bead expulsion time in vehicle- and DPA-treated mice (*n* = 10). Data are presented as mean ± SD. **P < 0.01 (**B**, paired 2-tailed *t* test; **H**, unpaired 2-tailed *t* test).

To identify the contraction-inhibiting substance produced by PIB, we extracted PIB culture supernatants with a methanol/H_2_O/dichloromethane reagent and separated the solution into 2 phases. The substances in the dichloromethane (nonpolar) phase showed sustained high activity, while those in the H_2_O/methanol (polar) phase had almost no activity (Figure 3, A and B). We then resolved the substances of the nonpolar phase by HPLC. In contrast to the LB medium control, the nonpolar phase of PIB culture supernatants produced a high additional peak about 10.2 minutes after elution (Figure 3C). Functional analysis showed that the substance within this peak had potent contraction-inhibiting activity, while other peaks had no or less activity (Figure 3D). Liquid chromatography–tandem mass spectrometry (LC-MS/MS) showed 3 unique fragments — with molecular weights of 329.2478, 285.2594, and 59.0146 Da — which were extracted as DPA by PeakView software (Figure 3E). We then measured the effect of pure commercial DPA (cDPA) on colonic contraction. 3 × 10^–6^ M cDPA inhibited contraction force by 20%, while 2.5 × 10^–4^ M cDPA almost completely inhibited the contraction (Figure 3, F and G). Using cDPA as a control, we quantified the level of DPA freshly produced in PIB culture supernatants (pDPA) and observed that pDPA concentrations ranged from 2.9 × 10^–6^ M to 5.7 × 10^–6^ M. The fresh pDPA was slightly more efficient in inhibiting contraction, which might be due to the partial activity loss of cDPA during production processes. To test the role of DPA in colonic contraction in vivo, we administered cDPA through the colon in mice, followed by glass bead insertion, and measured time to expulsion of beads. As expected, expulsion time was significantly reduced in comparison with that in the vehicle-treated group (vehicle 10.6 ± 0.7 minutes vs. DPA 14.7 ± 1.1 minutes, *P* < 0.01, Figure 3H). To determine whether dietary DPA inhibited colon function, we gavaged each mouse with 10 μL fish oil containing 200 μg DPA (equal to 60 softgels, or 600 mg DPA/60 kg body weight) and measured whole gut transit time. This high dose of dietary DPA did not alter the transit time (before gavage 128.4 ± 6.7 minutes vs. after gavage 132.5 ± 7.3 minutes, *n* = 10, *P* > 0.05).

**Figure 4:**
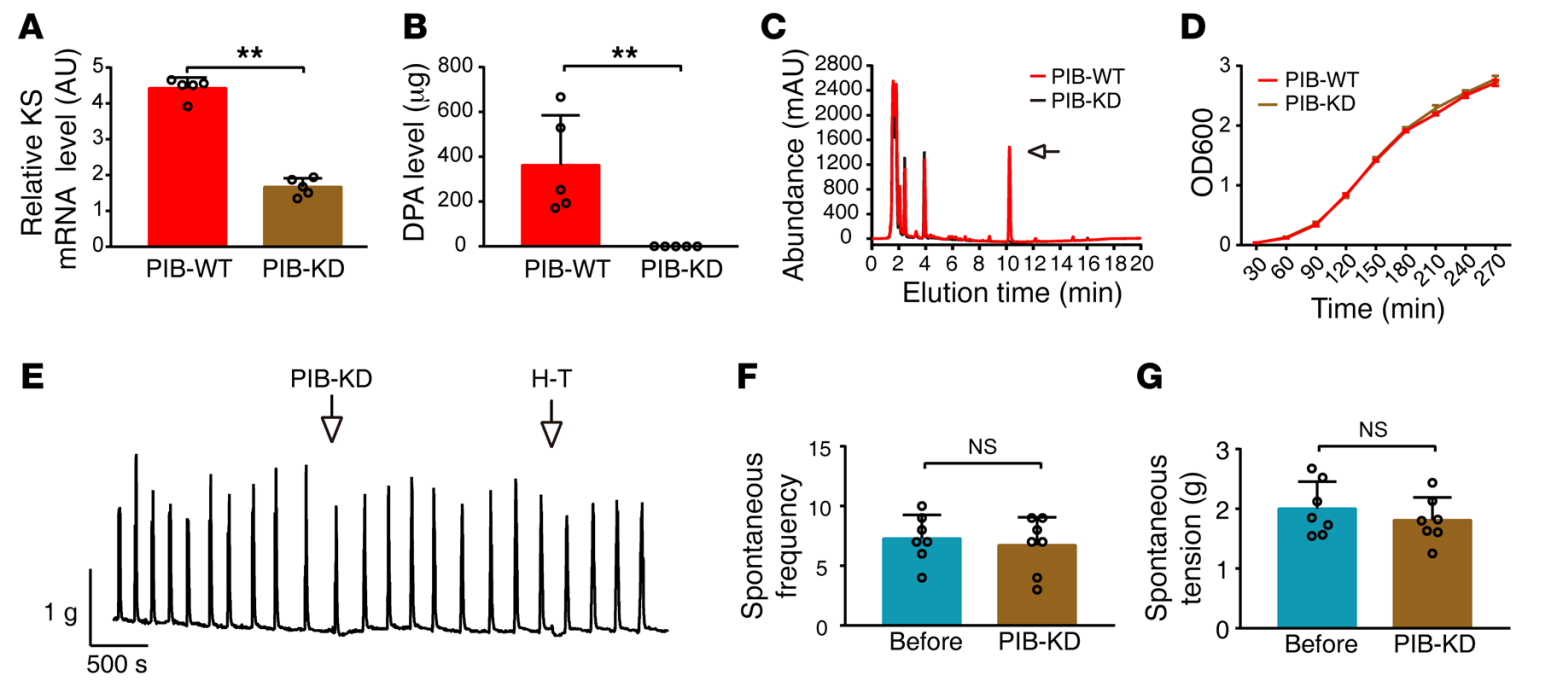
Knockdown of the KS gene disrupts the inhibitory effect of PIB on colon contraction. (**A**) KS mRNA level in the PIB-WT and PIB-KD strains was measured by qPCR in which the 16S rrs was used as an internal control (*n* = 5). (**B**) DPA levels within PIB-WT and PIB-KD culture supernatants were analyzed with LC-MS/MS (*n* = 5). (**C**)Chromatograph of the substances within the culture supernatants of PIB-WT and PIB-KD. The arrow indicates the chromatographic peak of DPA. The experiments were repeated at least 3 times. (**D**) Bacterial growth curves of PIB-WT and PIB-KD within 270 minutes (*n* = 5). (**E**) Representative contraction tracings of colons before and after treatment with PIB-KD culture supernatant. (**F** and **G**) Quantification of **E** (*n* = 7). The data are presented as mean ± SD. **P < 0.01 (**A** and **B**, unpaired 2-tailed *t* test; **F** and **G**, paired 2-tailed *t* test).

To test whether DPA was the only active substance in the PIB culture supernatant, we established a PIB variant expressing reduced DPA using a knockdown strategy (17). The synthesis of such polyunsaturated fatty acids (PUFAs) as eicosapentenoic acid (EPA), DPA, and docosahexenoic acid (DHA) in microorganism involves a polyketide synthase (PKS) pathway. This pathway is composed of acyltransferase, acyl carrier protein, malonyl coenzyme A transacylase, ketoacyl synthase (KS), ketoacyl reductase, dehydratase, enoyl reductase, and chain length factor domains (18). As EPA (C20) is the substrate of DPA (C22) and KS catalyzes carbon chain elongation from C20 to C22 (19), we considered the possibility that KS might be the essential target of DPA production. We used a CRISPR interference system targeting the KS gene and established a knockdown PIB variant (PIB-KD). Real-time PCR showed that the level of KS mRNA in PIB-KD was significantly reduced compared with that in WT PIB (PIB-WT) (Figure 4A). The DPA level was accordingly reduced about 1000-fold (Figure 4, B and C). As PIB-KD exhibited a growth curve comparable to that of the control (Figure 4D), KS knockdown did not significantly affect PIB’s physiological activity. The PIB-KD culture supernatant did not significantly inhibit spontaneous contraction of the colon (Figure 4, E–G). Collectively, our observations suggest that DPA is the key, if not the sole, active substance within the PIB culture supernatant in the context of inhibiting colon contraction.

### Oral administration of PIB is sufficient to colonize mice and induce constipation.

A specific measurement method was required to determine whether PIB could colonize mice. However, the challenge was how to identify a gene capable of distinguishing a PIB isolate from the large number of fecal microbes. We did not find a unique DNA region for PCR designation from noncoding RNAs or other genes by examining the whole PIB genome. This may be due to the fact that although the PIB genome contained 46 noncoding RNAs — many more than other *Shigella* strains — several other fecal bacterial genomes also had abundant noncoding RNA genes (e.g., the *E*. *coli* genome had 79 noncoding RNAs; Table 1). We then analyzed the SNPs within PIB coding regions. We identified a SNP candidate (g.331G→A) located at the sorbitol dehydrogenase gene that can be used as a specific marker in a PCR assay (Supplemental Figure 1A; supplemental material available online with this article; https://doi.org/10.1172/JCI150097DS1). We designated this assay as PCR PIB2013 because the sorbitol dehydrogenase gene was the 2013th gene within the PIB genome. To assess the sensitivity of PCR PIB2013, we mixed 1 to 1 × 10^6^ PIB with fresh healthy feces, cultured the mixture for 12 hours, and performed the assay. The results showed that PIB2013 was sensitive enough to detect a single PIB in feces (Supplemental Figure 1B). PIB detection of fecal bacteria from healthy volunteers revealed no positive signal (Supplemental Figure 1C), indicating the high specificity of PIB2013. We thus used this assay for subsequent detection.

**Figure 5:**
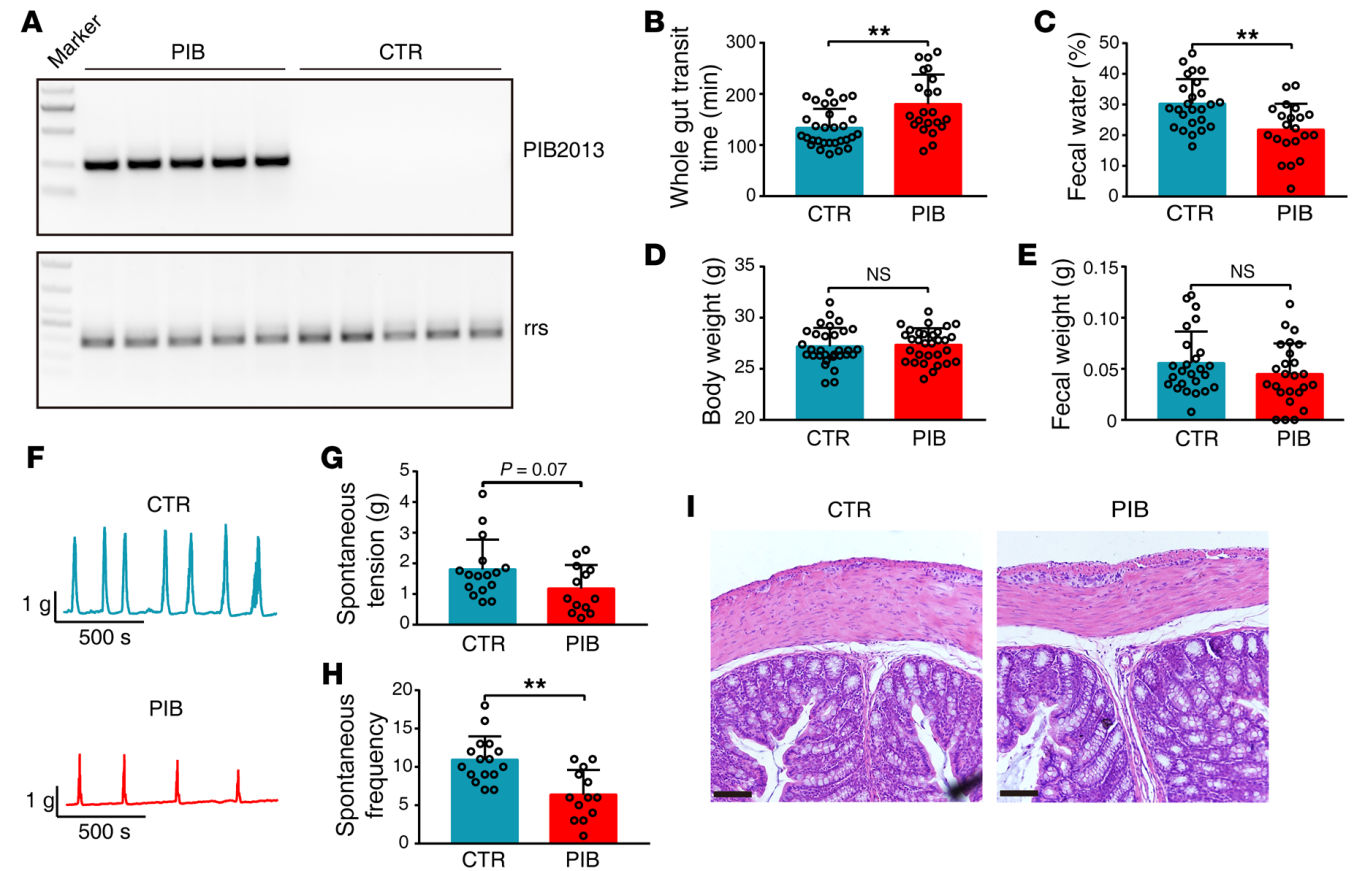
Oral administration of PIB is sufficient to colonize mice and induce constipation phenotypes. Introduction of 1 × 10^9^ CFU PIB (0.1 mL) per mouse was performed with gavage, and the constipation phenotypes were examined at week 9. (**A**) Fecal PIB was measured using PIB2013 (*n* = 5). 16S rrs was used as the internal control. CTR, control. (**B**) GTT (CTR, *n* = 30; PIB, *n* = 22). (**C**) Fecal water content (CTR, *n* = 25; PIB, *n* = 21). (**D**) Body weight (CTR, *n* = 30; PIB, *n* = 30). (**E**) Fecal weight (CTR, *n* = 25; PIB, *n* = 25). The data represent the summary from at least 3 independent experiments. Mice treated with LB were used as the control. (**F**) Representative colonic contraction tracings of PIB-treated and untreated mice. (**G** and **H**) Quantification of colonic peristaltic contraction in PIB-treated and control mice (CTR, *n* = 15; PIB, *n* = 13). (**I**) Representative histological morphology of colons from PIB-treated and control mice. The experiments were repeated at least 3 times. Scale bars: 100 μm. Data are presented as mean ± SD. **P < 0.01 (unpaired 2-tailed *t* test).

**Figure 6:**
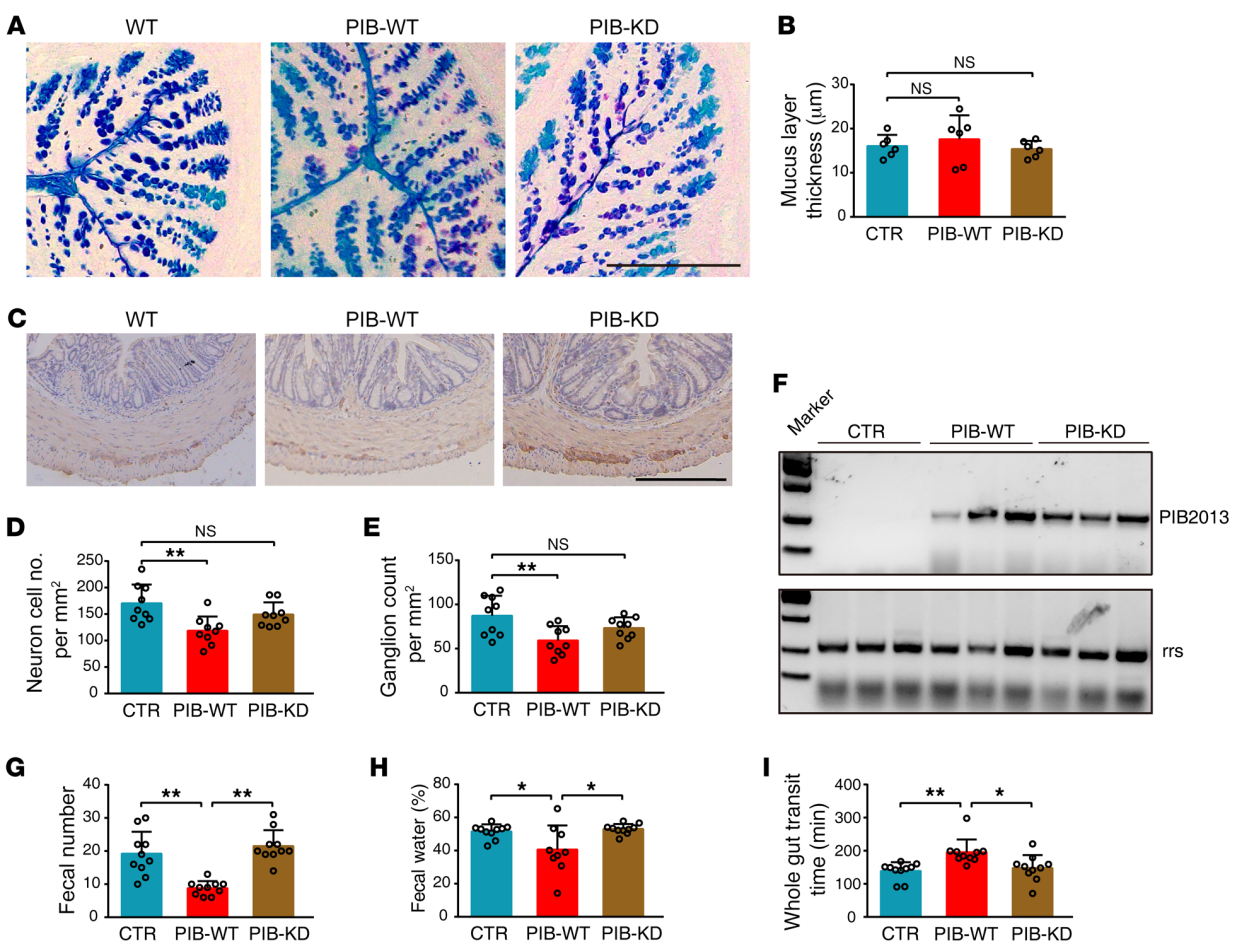
PIB-KD colonized mice but did not induce constipation. 1 × 10^9^ CFU PIB-KD or PIB-WT (0.1 mL) was administered orally to each mouse. (**A**) Typical immunochemistry section of mucus layers of colons from different groups of mice. (**B**) Quantification of mucus layer thickness (*n* = 6). (**C**) Representative immunochemistry results for colonic ganglia. (**D** and **E**) Quantification of ganglia and neurons (*n* = 9). (**F**) PIB2013 detection of fecal PIB-WT and PIB-KD in the stools. (**G**) Number of stools within 2 hours (*n* = 10). (**H**) Fecal water (*n* = 10, except PIB-WT *n* = 9). (**I**) GTT time (*n* = 10). Data are presented as mean ± SD. *P < 0.05, **P < 0.01 (1-way ANOVA Tukey’s multiple-comparison test). Scale bars: 200 μm.

To test whether PIB colonizes animals and induces constipation phenotypes, we gavaged approximately 1 × 10^9^ bacteria per mouse each week without antibiotics. After 8 weeks, PIB was detected in the feces. Figure 5A represents a typical PCR result for fecal PIB detection. PIB^+^ and control groups were examined for constipation phenotypes including gastrointestinal motility, fecal properties, and colon histology. The gastrointestinal transit time (GTT) of PIB-treated mice increased to 134% of that of the control (control 134 ± 7 minutes vs. PIB 180 ± 12 minutes, *P* < 0.01, Figure 5B), while fecal water content was reduced significantly (control 30% ± 2% vs. PIB: 22% ± 2%, *P* < 0.01, Figure 5C). Body weights and dried feces weights among these groups were comparable (Figure 5, D and E). In colons of PIB-treated mice, contraction amplitude and frequency were inhibited (Figure 5, F–H), while no inflammation or mucus layer alteration was observed (Figure 5I and Figure 6, A and B). However, we found a moderate reduction in enteric ganglia in the constipated (PIB-WT) but not the PIB-KD colons (Figure 6, C–E), implying that the enteric ganglion was a possible target of PIB. These results indicated that PIB colonized mice and induced constipation phenotypes. To determine whether these phenotypes were caused by DPA production by PIB, we gavaged approximately 1 × 10^9^ PIB-KD per mouse without antibiotics. After 8 weeks of gavage, PIB-KD was detected in the stool (Figure 6F), indicating that PIB-KD colonized mice as well. However, PIB-KD did not induce constipation phenotypes, as evidenced by the fact that there was no alteration of defecation frequency (control 19 ± 2 minutes vs. PIB-KD 22 ± 2 minutes, *P* > 0.05, Figure 6G), fecal water content (control 51.5% ± 1.4 % vs. PIB-KD 53.0% ± 1.0 %, *P* > 0.05, Figure 6H), or GTT (control 139.2 ± 8.3 minutes vs. PIB-KD 147.9 ± 12.4 minutes, *P* > 0.05, Figure 6I). In addition, the colonic mucus layer and enteric ganglia were similarly unaltered (Figure 6, A–E). This result indicates that DPA production by PIB was required for constipation.

### Epidemiologic distribution of PIB infection in IFC and non-IFC human populations.

We collected fecal bacteria from 3 groups, including healthy volunteers (*n =* 97), participants with IFC (*n =* 68), and participants without IFC (*n =* 83) (Table 2). The non-IFC group included 26 patients with laxative-sensitive constipation and 57 patients with ulcerative colitis with abnormal gut microbiomes (e.g., dysbacteria). All IFC patients were diagnosed at Jinling Hospital, Medical School of Nanjing University, and they strongly requested surgical procedures (10). The PIB2013 assay showed that all healthy volunteers and non-IFC patients were negative for fecal PIB, while 38 of 68 (55.9%) IFC patients were positive for fecal PIB. No age correlation was observed for PIB^+^ IFC patients. Moreover, the presence of PIB was not associated with disease history, treatment history, or syndrome severity (Table 3). In our study, the majority of IFC patients tested positive for fecal PIB, the presence of which may not be caused by an abnormal microbiome.

**Figure 7:**
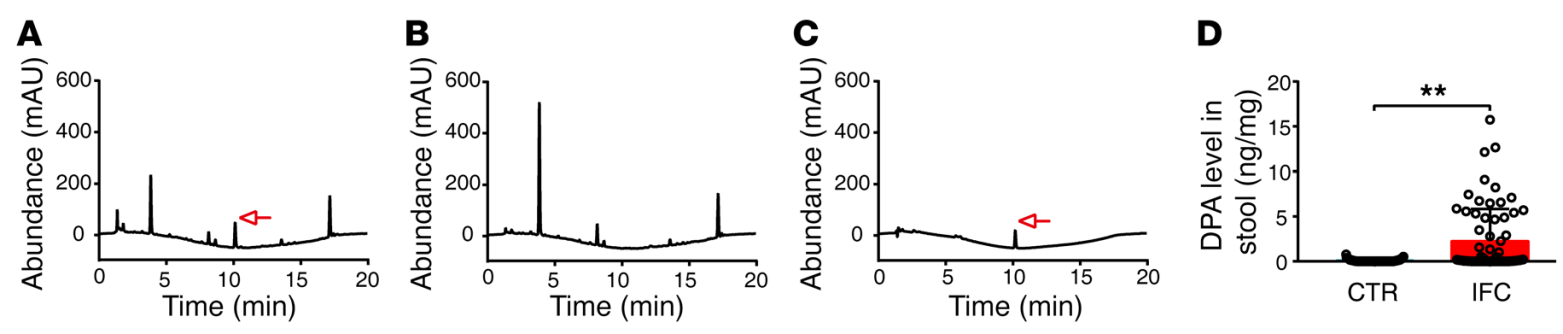
Fecal DPA levels are higher in patients with IFC. Stool specimens from participants with IFC and healthy volunteers underwent methanol/H_2_O/dichloromethane extraction and were sampled for HPLC analysis. (**A** and **B**) Representative chromatograms for fecal DPA in samples from the IFC and healthy groups. The arrow indicates a fecal DPA peak. (**C**) A liquid chromatogram of DPA standards (0.03125 ng) was performed to calculate DPA concentration in stool, indicated by an arrow. (**D**) Relative fecal DPA levels in the IFC patient and healthy groups (CTR, *n* = 97; IFC, *n* = 68). Data are presented as mean ± SD. **P < 0.01 (unpaired 2-tailed *t* test).

Given the finding in our study that IFC was caused by DPA-expressing PIB in the colon, a high level of DPA in IFC stool was expected. We thus analyzed stool DPA levels of 68 IFC patients and 97 healthy volunteers using HPLC. Of the IFC patients, 44.1% (30 of 68) showed a characteristic DPA peak, while none of the other IFC patients or the healthy volunteers (0 of 97) had an apparent DPA peak (Figure 7, A and B). Quantification by using cDPA as an external standard showed that the average DPA concentration of dried IFC stool was significantly higher than that of healthy volunteers (2.26 ± 0.43 ng/mg vs. 0.07 ± 0.01 ng/mg, *P* < 0.01) (Figure 7, C and D). The DPA concentrations in PIB^+^ wet stool were then calculated to be roughly 2.3 × 10^–6^ M. This result suggested that fecal DPA levels were elevated in IFC stool: 21 of 38 PIB^+^ IFC patients showed no DPA elevation, and 13 of 30 PIB^–^ IFC patients showed DPA elevation. About 25% (17 of 68) of IFC patients were PIB^+^ and had DPA elevation; 21 of 68 were PIB^+^ and DPA normal; 13 of 68 had DPA elevation and were PIB^–^; and 17 of 68 were PIB^–^ and DPA normal. This inconsistency regarding the presence of PIB and DPA production may have been due to their distribution within a stool sample. We indeed observed that different areas of the same stool sample showed differences in DPA level and the presence of PIB, indicating that PIB and DPA might not always be distributed uniformly within stools.

### Eliminating PIB could recover intestinal motility.

**Figure 8:**
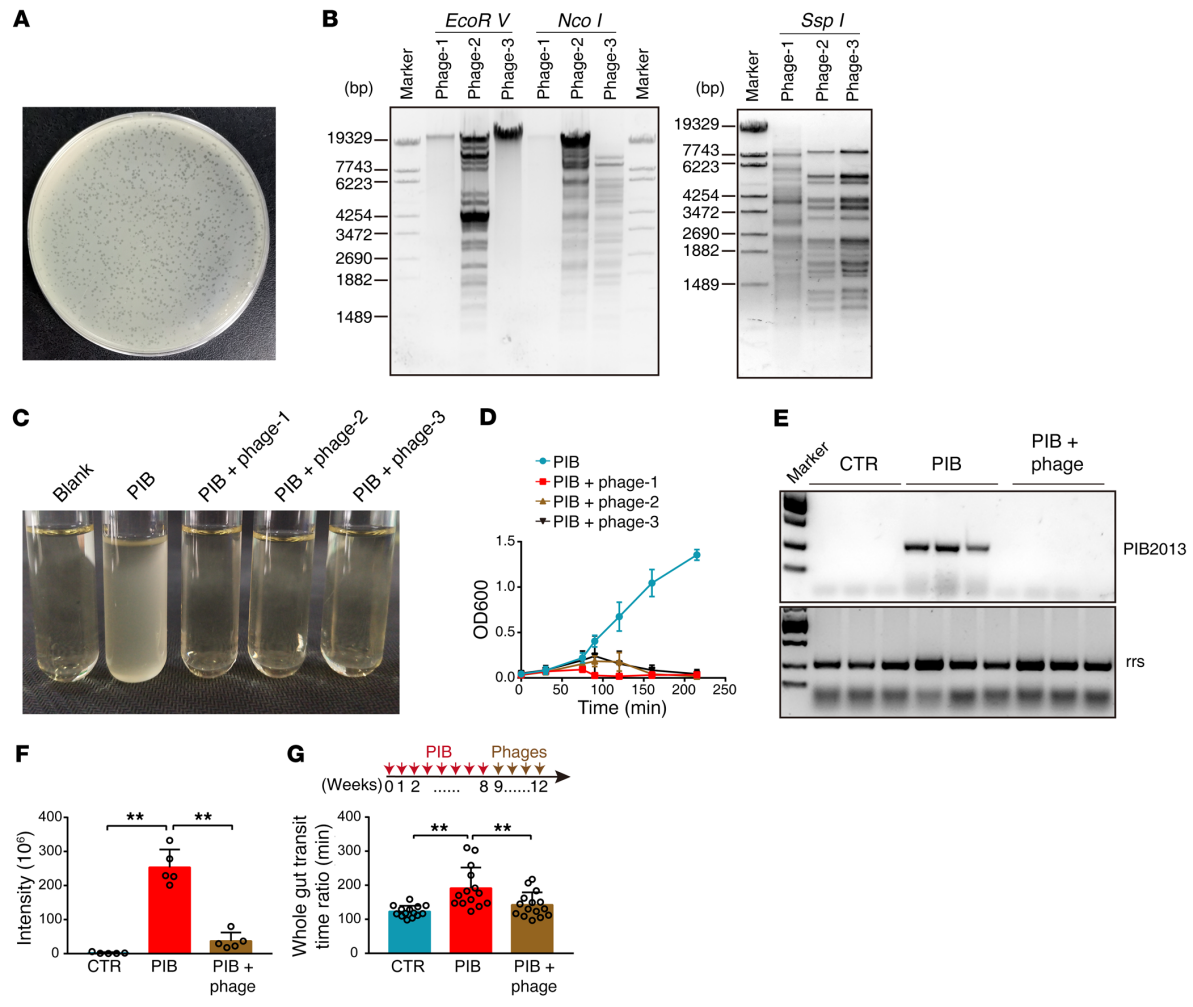
Treatment with bacteriophage against *Shigella* sp. PIB improves constipation symptoms. (**A**) Plaque morphology of an isolated *Shigella* sp. PIB phage from sewage water. (**B**) Restriction enzyme analysis for 3 phages. (**C**) Typical image of PIB medium after specific phage administration. Blank, LB only. (**D**) Growth curve of PIB with or without *Shigella* phages (*n* = 4). (**E**) Fecal PIB in the indicated groups of mice as assayed by PIB2013. (**F**) Fecal DPA levels in the indicated mouse groups (*n* = 5). (**G**) GTT time for mice with or without PIB-specific phages (CTR, *n* = 14; PIB, *n* = 14; PIB + phage, *n* = 15). Data are presented as mean ± SD. **P < 0.01 (1-way ANOVA Tukey’s multiple-comparison test).

To develop a method to eliminate PIB in vivo, we screened PIB-specific bacteriophages from sewage water and obtained about 100 bacteriophage colonies (Figure 8A). Restriction enzyme digestion analysis showed 3 different phage candidates (Figure 8B). We next analyzed 3 bacteriophage colonies and found that they all had potent PIB-lysing activity (Figure 8, C and D). After 4 weeks of administration of the mixture of phages 1, 2, and 3, 83.3% of constipated animals were fecal PIB^–^, while all the animals without treatment were PIB^+^. Importantly, the presence of PIB was not detected in the phage-treated mice even 6 months after treatment was withdrawn, indicating the phages eliminated PIB completely (Figure 8E). Consistent with this finding, application of the phages led to a significant reduction in the level of fecal DPA (Figure 8F). Notably, after administration of the phages, intestinal motility of the PIB^+^ constipated mice improved (PIB + phage 143 ± 10 minutes vs. PIB 192 ± 16 minutes, *P* < 0.05) (Figure 8G). This result showed that the bacteriophages not only efficiently decreased PIB colonization and DPA levels in the colon, but also reversed the constipation phenotype.

## Discussion

In this report, we identified a bacterial isolate, *Shigella* sp. PIB, from colons of patients with IFC. PIB inhibited gastrointestinal contraction by producing an unsaturated fatty acid, DPA. Because (a) oral administration of PIB was sufficient to establish colonization in mice and induce constipation phenotypes and (b) removal of PIB by specific bacterial phages significantly attenuated constipation, we propose that PIB is a causative pathogen of IFC. The clinical evidence that fecal PIB and DPA were found exclusively in patients with IFC also strongly supports this conclusion. As 75% of the IFC patients were either PIB^+^ or showed DPA elevation in stool, a majority of IFC cases might therefore be considered infectious diseases. As we found PIB to be a type of mucosal bacteria capable of persistently colonizing the colon, such a concept would explain the intractable nature of IFC. We note that 25% of IFC patients showed neither the presence of PIB nor fecal DPA elevation. This might be due to the following: (a) The IFC patients we selected might have had other disorders or had IFCs with other etiologies. (b) Fecal PIB was not detected due to improper sample collection (e.g., the collected stools were too dry or samples were from patients undergoing antibiotic treatment). (c) The PIB or PIB-produced DPA was not properly measured due to nonuniform distribution in the stool.

The identification of PIB has several potential clinical implications. First, because the healthy population and non-IFC patients are negative for fecal PIB and DPA, positivity could indicate the presence of IFC. The method of detecting fecal PIB and DPA presented in our study could be a reliable tool to identify PIB^+^ IFC patients within the larger population of individuals with constipation. Second, this bacterial entity could provide an opportunity to develop therapeutic avenues for IFC disease, e.g., screening for PIB-specific phages or use of sensitive antibiotics. Third, as PIB from the human colon colonized WT mice without germ-free treatment, there is risk for cross-species infection. However, this risk is likely low due to the low level of PIB in feces. Last, although the Rome or American Gastroenterological Association criteria have been widely used to clinically classify, diagnose, and treat constipation (20, 21), these criteria primarily rely on symptoms such as defecation frequency and abdominal pain. For example, a study showed that nearly 44% of individuals with FC had IBS-C symptoms, while 90% of IBS-C patients met FC criteria, indicating confusion of current clinical diagnoses (22). Identification of PIB could lead to reclassification of constipation to include an infectious category; classification of constipation as PIB^+^ or PIB^–^ could aid in patient diagnosis.

DPA is a member of the Omega-3 fatty acid family, which are known for cardiovascular protection. Omega-3 fatty acids are long-chain PUFAs including α-linolenic acid (ALA; 18:3n-3), stearidonic acid (SDA; 18:4n-3), eicosapentenoid acid (EPA, 20: 5n-3), DPA (22: 5n-3), and DHA (22: 6n-3), which were first found in marine animals (23). Omega-3 fatty acids may be also produced by microorganisms through the PKS system. Although most microorganisms have a PKS system, only marine microorganisms can produce omega-3 fatty acids (18). Recent observations suggest that nonmarine bacteria also produce omega-3 fatty acids when the synthase genes of marine bacteria (EPA synthase genes of *Photobacterium profundum* and DHA synthase genes of *Methylophaga marina*) are transferred into *E*. *coli* (19). We consider PIB to be a *Shigella* variant, and how it gained the ability to produce DPA remains unclear. There are at least 2 possibilities: (a) PIB obtained genes from marine organisms or derived from marine organisms. (b) PIB acquired gain-of-function mutations in the PKS system. Investigation of the origin of PIB and its epidemiologic distribution in the natural world warrants future study. Marine bacterial strains and other DPA-producing bacteria (e.g., *Aetherobacter* spp. SBSr002 and SBSr003) (24, 25) were not identified in the IFC patients, suggesting that characteristics besides DPA production (such as the capacity to colonize the colon) are required in order to cause constipation. Nevertheless, whether other DPA-producing bacteria can induce IFC requires further research.

The average concentration of fecal DPA in participants with IFC was 2.26 ng/mg for dried stool and about 2.3 × 10^–6^ M for wet stool. We accordingly found that 3 × 10^–6^ M DPA effectively inhibited colon contraction. However, a question remained whether dietary DPA could inhibit colon contraction and thereby increase the risk of constipation. In this study, we orally administered a high dose of dietary DPA to mice (30-fold higher than that recommended for a human adult) and did not observe an alteration of colon function. Thus, DPA affected colon motility and induced constipation only when applied through the colon in vitro but not through diet. This may be due to the fact that, similar to other PUFAs, dietary DPA is absorbed thoroughly in the small intestine (26). It is plausible that the fecal DPA we detected in the IFC patients was primarily produced by PIB in the colon rather than by food intake. The trace level of fecal DPA we detected in healthy volunteers may have been produced from body fluid or exfoliated intestinal cells.

In conclusion, we identified a pathogenic bacterium (*Shigella* sp. PIB) underlying IFC in human colons. This bacterium induced constipation by producing DPA. Measurements of fecal PIB and DPA could be reliable methods to identify IFC.

## Methods

### Specimens and fecal bacteria culture.

Participants eligible for inclusion in IFC were defined according to following criteria: (a) FC met Rome IV criteria; (b) slow colonic transit time was confirmed by double-contrast barium enema, anorectal manometry with electromyography, and defecography; (c) life quality was severely threatened and could not be improved by conservative treatments such as high-fiber diet, increased water consumption (1.5 L/d), and laxatives; and (d) patients expressed the desire to undergo surgery (10). The IFC patients who requested surgery constituted our research group. The exclusion criteria included presence of tumor disease, inflammatory bowel disease, and nervous system disease. All patients reported disease, medication, and allergy history. Eighty-three non-IFC patients were hospitalized in the Department of Gastroenterology and Hepatology, Jinling Hospital, Medical School of Nanjing University; of these 57 were diagnosed with ulcerative colitis and 26 were self-diagnosed with constipation but not clinically diagnosed with IFC.

Fresh ascending colon segments were collected from IFC patients by subtotal colectomy with an improved Duhamel procedure at Jinling Hospital (10, 27). Colonic mucosa was washed with sterile PBS at least 5 times, then dissociated in PBS solution with sterile instruments and centrifuged at 1500*g* for 10 minutes. The supernatant was plated onto blood agar plates (1% peptone, 0.3% beef extract, 0.5% NaCl, 1.5% agar, and 5% sterile defibrinated sheep blood, pH 7.3 ± 0.1) and cultured for 12–16 hours at 37°C. The bacterial colonies were reinoculated individually into culture tubes with 3 mL LB medium (1% tryptone, 0.5% yeast extract, and 1% NaCl, pH 7.3 ± 0.1). The culture supernatants were collected and subjected to ex vivo contraction measurement.

### Bacterial screening.

Colon segments (5 mm in length) were isolated from 8- to 10-week-old C57BL/6J mice and mounted on a force transducer (MLT0201, ADInstruments) connected with a PowerLab recording device (ML785, ADInstruments) for monitoring of isometric contraction. The segment was suspended in the longitudinal axis of the muscle with 0.5 g resting tension in an organ bath (37°C) containing HEPES-Tyrode (H-T) buffer (137 mM NaCl, 2.7 mM KCl, 1.0 mM MgCl_2_, 1.8 mM CaCl_2_, 10 mM HEPES, and 5.6 mM glucose, pH 7.4) with a continuous pure oxygen supply (28). Thirty minutes after equilibration, fresh bacterial culture medium was added to the bath (5 mL H-T). Each medium was prepared by culturing a single bacterial colony for 12 hours when OD_600_ reached 2.5. Thirty minutes after tension recording, the bath buffer was replaced with fresh H-T solution in order to recover the segment. Usually, contractility of the segment could completely recover. When incubated with the PIB culture supernatant, the segment was recovered substantially (~50%) but not completely. This might be due to the presence of residual DPA infused into the colon tissue. For the screening experiment with jejunum, the procedures were identical, except 0.2 g resting tension was applied.

### GTT analysis.

Mice were fasted overnight, then administered intragastrically a 100 μL test meal (5% Evans blue and 1.5% methyl cellulose). Next, the mice were kept in individual cages, and the time from food administration to the time when blue feces was first observed was recorded as the GTT (29). Mice that did not expel blue feces within 6 hours were not included in the results. For a functional test with fish oil, GTT analysis was performed after 90 minutes with fish oil (Pharmatech Co., Norsk OMEGA3) gavage.

### Frequency of defecation and water content.

Mice with free feeding were kept in a quiet environment for 12 hours, and their feces at the last 2 hours were collected and quantified. The number of fecal pieces collected per the 2-hour period was the frequency of defecation. Fecal water content was weight change from fresh to dry. Urine-soaked stool was not included in the fecal water test.

### Phylogenetic tree analysis.

PCR amplification of the PIB 16S ribosome RNA coding gene was performed with a pair of primers: 27F, 5′-AGAGTTTGATCCTGGCTCAG-3′ and 1492R, 5′-GGTTACCTTGTTACGACTT-3′. PCR amplification was performed with 2 × Phanta Master kit (P511-01, Valzyme Co.) in a 50 μL reaction, including 25 μL 2 × Phanta Master Mix, 2 μL 27F primer (10 μM, synthesized by Genscript), 2 μL 1492R primer (10 μM, synthesized by Genscript), 1 μL bacterial culture mixture (OD_600_ ≥ 2.5), and 20 μL ddH_2_O. Thermal cycling consisted of an initial denaturation step (95°C, 3 minutes), followed by 35 cycles of denaturation (95°C, 10 seconds), annealing (60°C, 10 seconds), and extension (72°C, 45 seconds). The final extension was 72°C for 10 minutes. We used ClustalW to sequence, align, and cluster the PCR product with other 16S rrs (bacteria and archaea). The phylogenetic tree was constructed with Molecular Evolutionary Genetics Analysis software (version 7.0.14, MEGA).

### Genome sequencing and assembly.

PIB genomic DNA was sequenced with combined next-generation (Illumina HiSeq 4000) and SMRT (PacBio RS II) sequencing at the Beijing Genomics Institute (BGI). Library construction, self-correction, and data analysis were performed in accordance with the manufacturer’s protocols. The genomic sequence was deposited in GenBank, with BioProject accession number PRJNA792051.

### Genome component prediction and annotation.

Prediction of a protein-coding sequence was performed by GLIMMER3 (http://ccb.jhu.edu/software/glimmer/index.shtml) with hidden Markov models. Analysis of transfer RNA (tRNA), rRNA, and bacterial small RNA (sRNA) was conducted with 3 databases (tRNAscan-SE [http://gtrnadb.ucsc.edu/], RNAmmer [https://services.healthtech.dtu.dk/service.php?RNAmmer-1.2], and Rfam [http://rfam.xfam.org/]). The tandem repeats were identified by Tandem Repeats Finder (http://tandem.bu.edu/trf/trf.html), and the minisatellite and microsatellite DNAs were represented by the number and length of repeat units. The genomics lands, prophage regions, and CRISPR regions were predicted with the Genomic Island Suite of Tools (30), PHAge Search Tool (PHAST, http://phast.wishartlab.com/), and CRISPRFinder (https://crisprcas.i2bc.paris-saclay.fr/CrisprCasFinder/Index).

Coding sequence (CDS) annotation was analyzed by BLASTP (https://blast.ncbi.nlm.nih.gov/Blast.cgi) and 7 databases: Kyoto Encyclopedia of Genes and Genomes (KEGG; https://www.genome.jp/kegg/); Clusters of Orthologous Groups (COG; https://www.ncbi.nlm.nih.gov/research/cog-project/); RefSeq Non-Redundant Proteins (NR) database (https://www.ncbi.nlm.nih.gov/refseq/about/nonredundantproteins/); Swiss-Prot (https://www.expasy.org/resources/uniprotkb-swiss-prot); Gene Ontology (GO; http://geneontology.org/); UniProtKB/TrEMBL (http://www.bioinfo.pte.hu/more/TrEMBL.htm); and EggNOG (http://eggnog5.embl.de/#/app/home). Virulence factors and resistance genes were recognized by the Virulence Factors of Pathogenic Bacteria (VFDB; http://www.mgc.ac.cn/VFs/) database and Antibiotic Resistance Genes Database (ARDB; https://ardb.cbcb.umd.edu/), respectively. Type III secretion system effector proteins were identified by EffectiveT3 (https://effectors.csb.univie.ac.at/method/effectivet3).

### Diagnostic detection of PIB in human and mouse stool.

Standardized protocol and kits for collecting stool were provided to study participants as described previously (31). The volunteers collected samples at home and delivered them to the laboratory at the Medical School of Nanjing University. About 1 g of stool sample was vortexed with sterile LB medium and subjected to a brief centrifugation (1500*g* for 10 minutes). The resultant supernatant was cultured in 3 mL LB medium overnight at 37°C. By examining the genomic sequence of PIB, we designed various primers for the PCR assay. We optimized a pair of primers for PIB detection (PIB2013: F, 5′-TGGTTTTGAATCAGGCCCGT-3′ and R, 5′-GTAGCTCCCACGCATTTGCA-3′). PCR amplification was performed in a 20 μL reaction containing 10 μL 2 × Taq Master Mix (Dye Plus, Valzyme Co.), 0.8 μL PIB2013 primer F and PIB2013 primer R (10 μM, Genscript Co.), 1 μL bacterial culture, and 7.4 μL ddH_2_O. Thermal cycling consisted of an initial denaturation step (94°C, 5 minutes), followed by 35 cycles of denaturation (94°C, 30 seconds), annealing (62°C, 30 seconds), and extension (72°C, 30 seconds). Final extension was 72°C for 5 minutes. The PCR product was resolved by agarose gel (1.5%) electrophoresis and validated by sequencing.

### Sample preparation for HPLC and LC-MS/MS.

Bacterial culture medium (15 or 50 mL) was equally divided into 10 tubes and then lyophilized by a SpeedVac machine (Thermo Fisher Scientific). The dried medium was extracted thoroughly by a method described previously (32). Briefly, 410 μL precooled methanol and 210 μL water were mixed with the medium, followed by addition of 140 μL precooled dichloromethane. After thorough vortex for 2 minutes, the mixture was further extracted with 140 μL dichloromethane and 210 μL water and incubated on ice for 20 minutes. By centrifuging for 10 minutes (13,000*g*, at 4°C), the samples were separated into 2 phases: the polar phase at the upper phase and the nonpolar phase at the lower phase. The solutions of both phases were collected separately and lyophilized with a SpeedVac machine (Thermo Fisher Scientific). The substances in the nonpolar phase were dissolved by 30 μL DMSO or the HPLC buffer of mobile phase A (acetonitrile/methanol/water containing 10 mM ammonium formate). 20 μL samples were applied for HPLC or LC-MS/MS analysis. To prepare the samples from stools, we collected fresh stool (~20 mg for mice and ~300 mg for participants), which we dried (~6 mg for mice and ~20–100 mg for participants) and extracted with 410 μL methanol/210 μL water/140 μL dichloromethane using the same procedure as above. 20 μL of the resultant samples in the nonpolar phase were subjected to HPLC or LC-MS/MS analysis.

### HPLC.

Chromatographic separation was achieved at 25°C on an Agilent 1200 with a column (C18; 150 × 4.6 mm; Yiliteng) at a flow rate of 1 mL/min. Mobile phase A was acetonitrile/methanol/water containing 10 mM ammonium formate (1:1:1, v/v/v), and phase B was acetonitrile/isopropanol (1:1, v/v). The gradient elution was 0–1 minutes, 15% B; 1–5 minutes, 50% B; 5–15 minutes, 95% B; 15–20 minutes, 95% B. Twenty elution fractions were collected and dried with a SpeedVac machine, then subjected to activity measurement and LC-MS/MS analysis. According to the proportional relationship of mAU value and DPA concentration, we quantified human DPA in dry stool.

### LC-MS/MS analysis and data processing.

The collected fraction of HPLC was sampled and analyzed by hybrid quadrupole–TOF (AB SCIEX Triple TOF 4600 instruments) LC-MS/MS detection with a column (Phenomenex Accucore C18, 150 × 2.1 mm, 2.6 μm). The mobile phases were identical to that of HPLC. The gradient elution was 0–1 minutes, 20% B; 1–4 minutes, 60% B; 4–10 minutes, 70% B; 10–15 minutes, 95% B; 15–20 minutes, 95% B. Mass spectrometry analysis was performed using a DuoSpray Ion Source in the negative ion modes. The TOF MS parameters were as follows: auxiliary air pressure, 55 psi; atomization gas pressure, 55 psi; air curtain gas, 35 psi; source temperature, 600°C; voltage floating, 4500 V. Mass range parameters were as follows: declustering potential, –80 eV; collision energy, –10 eV; start mass, 200 Da; end mass, 1300 Da; accumulation time, 200 ms. The TOF MS/MS parameters were the same as those of TOF MS. Mass range parameters were as follows: declustering potential, –80 eV; collision energy, –40 eV; collision energy spread, 20 eV; ion release delay and width, 30 and 15; start mass 50 Da; end mass, 1250 Da; accumulation time, 65 ms. Compound identities were extracted by the instrument software PeakView (Sciex). The signal intensity reflects the sample’s DPA concentration.

### Histological analysis.

For enteric ganglia staining, the colon segments (about 0.5 cm long) were isolated from mice and fixed overnight with 4% paraformaldehyde in PBS. Then the tissues were embedded in paraffin, cut into 6-μm-thick sections, and dewaxed with following protocol: xylene 10 minutes, xylene 10 minutes, 50% xylene/50% ethanol 2 minutes, 100% ethanol 2 minutes, 95% ethanol 2 minutes, 85% ethanol 2 minutes, 75% ethanol 1 minute, 50% ethanol 1 minute, water 1 minute. The section underwent antigen recovery by Citrate Antigen Retrieval Solution (E673001, Sangon Biotech Co.) and immunohistochemistry, which was performed according to the UltraSensitive SP IHC Kit (KIT-9720, MXB Biotechnologies Co.) manual. The colonic ganglia were visualized by staining with anti-PGP9.5 antibody (Abcam, ab8189, 1:5000), followed by nucleus staining with hematoxylin.

For immunohistochemical analysis of the mucus layer, colons (about 0.5 cm) were isolated and immediately fixed in Carnoy’s solution (R23046, Shanghai Yuanye Bio-Technology Co.) for 24 hours. Tissues were then washed by methanol (2 × 30 minutes), ethanol (2 × 15 minutes), ethanol/xylene (1:1; 15 minutes), xylene (15 minutes), and liquid paraffin (4 × 1 hour). Finally, the colon was embedded in paraffin and cut into 6-μm-thick sections. Next, the mucus layer was visualized by periodic acid–Schiff–alcian blue staining solution according to the manual for the AB-PAS kit (R20530, Shanghai Yuanye Bio-Technology Co.).

### Colonic transit assay.

Colonic transit was measured by monitoring bead expulsion time as described previously (33, 34). Eight-week-old mice were anesthetized by light ether prior to intracolonic administration with 100 μL DPA solution (300 mM in olive oil) or vehicle (olive oil) by catheter. Five minutes later, a glass bead (3 mm diameter) was inserted into the distal colon with a glossy glass rod (about 2 cm depth). The mice were placed in individual cages, and the time of bead expulsion was recorded.

### Construction of a Shigella sp. PIB mutant with knockdown expression of KS.

The CRISPR interference system was used to generate a mutant *Shigella* sp. PIB with knockdown of KS. The pdCas9 vector (Addgene, 44249) was used as a vector backbone. A fragment containing a J23119 promotor (5′-TTGACAGCTAGCTCAGTCCTAGGTATAATACTAGT-3′) and a guide DNA (gDNA) of KS (5′-TTACATTAAATATTACCGACTGG-3′) plus an sgRNA coding template was inserted upstream of the dCas9 coding sequence through a BglII site. The resultant vector (J23119 + KS gDNA + sgRNA coding DNA + J23119 + dCas9) persistently expressed the KS gDNA and dCas9, decreasing KS gene expression in bacteria. The KS gene knockdown bacterium (PIB-KD) was generated by electrotransformation (1800 W, 25 μF, 200 Ω, Bio-Rad) with the vector and selection with chloramphenicol (10 μg/mL). To verify the knockdown efficiency, the total RNA of the mutant bacterium was isolated with a Bacteria RNA Extraction Kit (R403-01, Vazyme Co.) and reverse transcribed to cDNA (Hiscript Q RT SuperMix for qPCR [+gDNA wiper] kit, R123-01, Vazyme Co.). The expression of the KS gene was quantified with a Taq Pro Universal SYBR qPCR Master Mix kit (Q712-02, Vazyme Co.). The expression of 16S rDNA was used as internal control.

### Bacteriophage isolation and administration.

*Shigella* sp. PIB phages were isolated from raw sewage of the Chuhe River in Nanjing. 50 mL raw sewage was centrifuged, and the supernatant was filtered with a 0.22 μm syringe filter. We mixed the filtrate and *Shigella* sp. PIB, then added in LB medium at the top (0.7% agar) and poured on LB solid medium (1.5% agar) to culture overnight at 37°C. Phages were selected from the resulting plaques and purified with repetitive inoculation and selection for 3 generations. The phage was stored in SM solution (0.58% NaCl, 0.2% MgSO_4_, 1% gelatin, 5 M Tris-Cl [pH 7.5]).

### Animals.

All mice were 8- to 10-week-old male C57BL/5J mice from the National Resource Center of Model Mice (NRCMM) of China, which were kept in a 12-hour light/12-hour dark cycle environment, with light turned on at 8:00 am. Mice had full access to food and water, except in the GTT experiment.

For bacterial colonization experiments, the WT or KD PIB (1 × 10^9^ CFU/100 μL) was gavaged into each mouse once a week; the same amount of LB (100 μL) was given as a control. Two months later, the indicated measurements were taken. The phage treatment assay was only done in the PIB^+^ group. 100 μL (5 × 10^8^ PFU/100 mL) phage solution was gavaged into each PIB^+^ mouse for 1 month.

### Statistics.

All data are presented as mean ± SD. Differences between 2 groups (2-tailed *t* test) or 3 groups (1-way ANOVA Tukey’s multiple-comparison test) were analyzed with GraphPad Prism version 7. A *P* value less than 0.05 was considered significant.

### Study approval.

All animal experiments were approved by the Animal Care and Use Committee of the Model Animal Research Center of Nanjing University, which is a member of the Association for Assessment and Accreditation of Laboratory Animal Care (AAALAC). Testing of human samples was approved by the Experimentation Ethics Review Committee of Jinling Hospital, Medical School of Nanjing University (2020NZKY-008-01). All participants were aware of the aim of this study, and written informed consent was received from participants or their legal guardians.

## Author contributions

XC and MSZ designed the research studies. XC, TTQ, YW and LYX performed experiments and acquired data. XC, JS, ZHJ, WZ, TT, YWZ, LSW, YQL, and YYZ analyzed results. XBF, FYW, NL, HQC, GHZ, and JJ collected and analyzed clinical samples. XC, JZ, XNZ, JJ, and MSZ wrote the manuscript. XNZ, JJ, and MSZ conducted the experiments.

## Supplementary Material

Supplemental data

## Figures and Tables

**Table 2:**
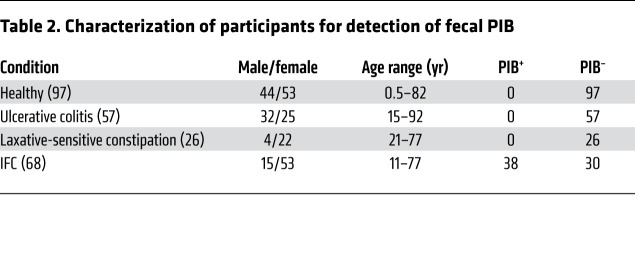
Characterization of participants for detection of fecal PIB

**Table 3:**
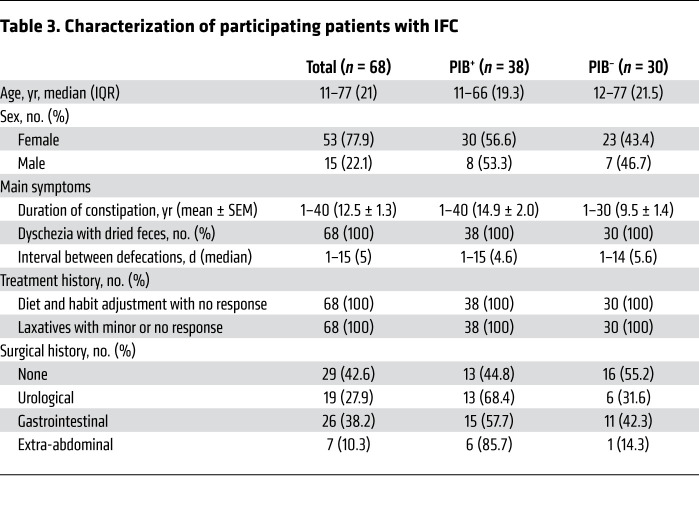
Characterization of participating patients with IFC

**Table 1:**
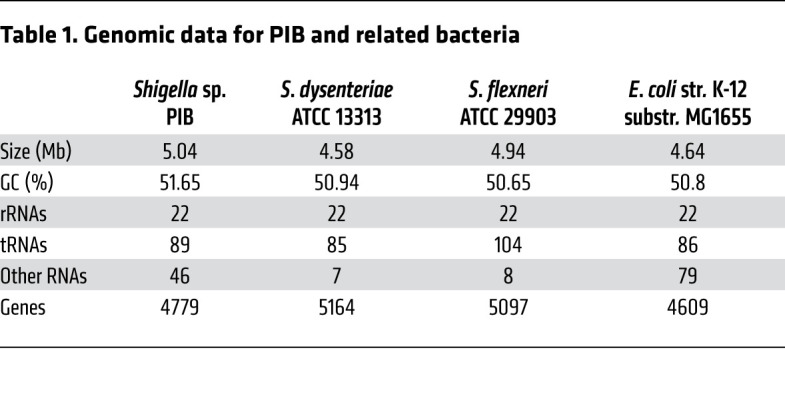
Genomic data for PIB and related bacteria

## Version 1 05/26/2022

In-Press Preview

## Version 2 07/15/2022

Electronic publication
