# Supplementary material for: A Shigella species variant is causally linked to intractable functional constipation
Source: J Clin Invest. 2022 Jul 15;132(14):e150097. doi: 10.1172/JCI150097 (PMC9282927; doi:10.1172/JCI150097)
Supplement: Supplemental data [file jci-132-150097-s056.pdf]

A

GCAAATATAACATCTGCCCGAACGTTGATTTTATGGCGACACAACCTAACTACCGCGGCGCATTAACG
CACTATCTGTGTCATCCGGAGAGCTTTACTTACAAACTGCCCGACAATATGGACACGATGGAAGGGGC
GCTGGTGGAGCCTGCCGCAGTCGGGATGCATGCCGCGATGCTGGCAGATGTTAAACCGGGTAAGAAGA
TAATTATTCTGGGAGCGGGTTGTATTGGTTTGATGACGTTGCAAGC

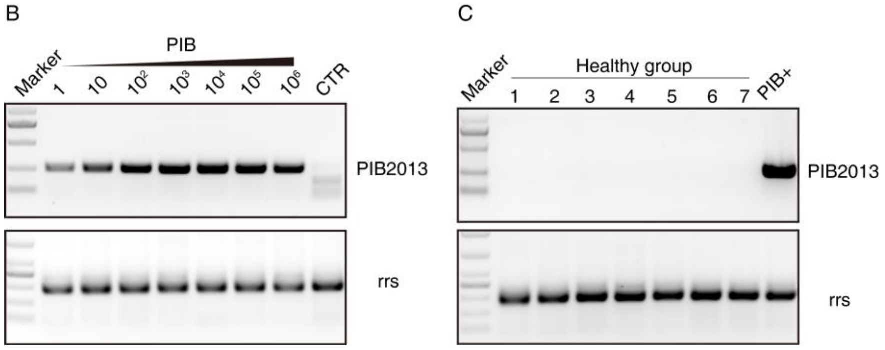

Supplemental Figure 1. PIB2013 specifically detected PIB presence from stool. Different amount of PIB bacteria were mixed with stool from a healthy adult (CTR) and the resultant bacterial mixtures were subjected to PIB2013 assay. (**A**) The target sequence of PIB genome for PIB2013 assay. (**B**) A typical PCR assay for PIB-containing samples (PIB+). (**C**) A typical PCR assay for CTR samples. rrs was used as the internal control.
